# Designing smartphone-based cognitive assessments for schizophrenia: Perspectives from a multisite study

**DOI:** 10.1016/j.scog.2025.100347

**Published:** 2025-02-05

**Authors:** Aishwarya Raje, Abhijit R. Rozatkar, Urvakhsh Meherwan Mehta, Ritu Shrivastava, Ameya Bondre, Manaal Amir Ahmad, Anshika Malviya, Yogendra Sen, Deepak Tugnawat, Anant Bhan, Tamonud Modak, Nabagata Das, Srilakshmi Nagendra, Erlend Lane, Juan Castillo, John A. Naslund, John Torous, Soumya Choudhary

**Affiliations:** a All India Institute of Medical Sciences, Bhopal, India; b National Institute of Mental Health and Neurosciences, Bengaluru, India; c Beth Israel Deaconess Medical Center, Harvard Medical School, Boston, USA; d Sangath, Bhopal, India; e Department of Global Health and Social Medicine, Harvard Medical School, Boston, USA

**Keywords:** Schizophrenia, Digital phenotyping, Cognition, Digital technology

## Abstract

**Introduction:**

Cognitive deficits represent a core symptom of schizophrenia and a principal contributor to illness disability, yet evaluating cognition in routine clinical settings is often not feasible as cognitive assessments take longer than a standard doctor's visit. Using smartphones to assess cognition in schizophrenia offers the advantages of convenience in that patients can complete assessments outside of the clinic, temporality in that longitudinal trends can be identified, and contextuality in that cognitive scores can be interpreted with other measures captured by the phone (e.g. sleep). The current study aims to design a battery of cognitive assessments corresponding to the MATRICs Consensus Battery of Cognition (MCCB), in partnership with people living with schizophrenia.

**Methodology:**

Focus group discussions (FGDs) and interviews were conducted with people diagnosed with schizophrenia across three sites (Boston, Bhopal, and Bangalore) to help design, refine, and assess the proposed smartphone battery of cognitive tests on the mindLAMP app. Interviews were conducted between December 2023 and March 2024. Inductive thematic analysis was used to analyze data.

**Results:**

Participants found the app and its proposed cognitive assessments to be acceptable, helpful, and easy to use. They particularly found the gamified nature of the cognitive tests to be appealing and engaging. However, they also proposed ways to further increase engagement by including more information about each cognitive test, more visual instructions, and more information about scoring. Across all sites, there were many similarities in themes.

**Discussion & conclusion:**

People living with schizophrenia, from different sites in the US and India, appear interested in using smartphone apps to track their cognition. Thematic analysis reinforces the importance of feedback and data sharing, although this presents a challenge, given the novel nature of smartphone-based cognitive measures that have not yet been standardized or validated.

## 1 Introduction

The intersection of technology and mental healthcare has seen significant advancements in recent years, particularly in developing and deploying mobile applications designed to support individuals living with psychiatric conditions (Chivilgina et al., 2020; Lecomte et al., 2020). As the prevalence of smartphone ownership increases globally, using apps to improve mental health becomes more feasible. Digital systems are already able to provide scalable support for people living with schizophrenia (Asher et al., 2018), and this is of critical value in low- and middle-income countries like India (Farooq et al., 2016; Gureje and Oladeji, 2022) where care options are limited.

To date, most digital health solutions in schizophrenia have focused on symptom reduction through offering app-based interventions. Such novel digital approaches for people with psychosis are increasingly proposed and assessed (Ben-Zeev et al., 2019; Torous et al., 2021). Recent reviews have highlighted the variety of these digital mental health tools for people with schizophrenia and their acceptability in global settings (Smith et al., 2024; Chukka et al., 2023). However, the majority of research has not focused on the domain that can contribute significantly to disability: cognition (Patel et al., 2014). While Lavigne et al. (2022) provide a thorough review of various approaches to remotely testing cognition in psychiatric populations, the authors highlight the absence of a “gold standard” remote battery for comprehensive cognitive assessment.

An international project funded by Wellcome Trust, the Improving Cognitive and Functional Outcomes in People Experiencing, or at Risk of Psychosis, “Scalable Digital Phenotyping of Momentary Cognition in Early Course Psychosis” endeavors to address this gap in the existing literature. The study targets to adapt the MATRICS framework (widely recognized as the gold standard for cognitive assessment) into digital cognitive assessment games in the MindLAMP app. The project aims to design a battery of smartphone assessments designed to measure the cognitive domains most impacted in early course schizophrenia, refine that battery in pilot testing, and assess the potential of these cognitive and contextual momentary performance metrics against functional outcomes with personalized scoring controlling for differing environments and circadian rhythms. The project builds on the mindLAMP open-source platform and mobile application (Torous et al., 2019), which has been used in prior international research to study digital phenotyping markers of relapse with passive smartphone data. This is a multi-site study across Boston (USA), Bangalore (India), and Bhopal (India) that explores the needs of individuals with schizophrenia and their families, emphasizing the potential of technology to build trust, maintain engagement, and ensure efficacy in future clinical applications. These sites were selected to represent diverse cultural contexts and varying levels of technological integration in mental health care.

Involving persons with lived experience and the target app users across all stages of development is vital to ensuring that digital mental health interventions are acceptable, usable, and effective (Smith et al., 2024). Studies that look at co-creating digital interventions with people with lived experiences in schizophrenia (Allan et al., 2019; Berry et al., 2020; Realpe et al., 2020; Rodriguez-Villa et al., 2021) highlight the value of this approach. Thus, this study focuses on the perspective of people with lived experience in evaluating and designing new features for smartphone-based cognitive monitoring using the mindLAMP app in schizophrenia.

## 2 Objective

The primary objective of this first phase of the study was to design a new suite of cognitive assessments in partnership with people living with schizophrenia. The insights derived from this study will be used to inform iterative development of the app, with a focus on the adapted cognitive games, ensuring it meets the needs of its intended users. By understanding the perspectives of stakeholders from multicultural backgrounds, this current study seeks to enhance the app's utility and acceptance, ultimately supporting the goal of providing equitable and effective mental health care through innovative digital solutions.

## 3 Methods

Interviews and focus group discussions were conducted to gather feedback regarding the features and functionality of the mindLAMP app. Feedback was obtained by following a semi-structured topic guide (Appendix 1) based on the objective of the study and informed by previous work on FGDs conducted on the use of mindLAMP app. The topic guide aimed to explore attitudes and experience on the following themes: (a) general phone and app use in daily life; (b) use of healthcare apps; and (c) feedback on the cognitive tests on the mindLAMP app. These topics were designed to explore user experiences, identify potential barriers to adoption, and gather suggestions for improving the app's functionality including recommendations for engagement strategies. The three sites for the study included various demographics, aiming to make the app acceptable to a broader cultural audience to achieve wider reach. The focus group discussions and individual interviews were conducted between December 2023 and March 2024. Ethical approval was obtained at each site and written informed consent was obtained from all participants.

### 3.1 MindLAMP app

MindLAMP app is an open-source research app designed to enhance mental health care through personalized interventions and digital phenotyping (Chang et al., 2023). The app has 4 main sections: Learn, where users can access helpful tips and resources; Assess, where they can complete surveys and play various cognitive games; Manage, where they can do meditation and breathing exercises; and Portal, where they can view their data. With permission from the user, the app can also collect sensor data such as GPS and accelerometer from the smartphone, which allows for real-time monitoring of mental health symptoms and behavior. One of the significant features of mindLAMP is the integration of cognitive training tools aimed at measuring attention and memory (Vaidyam et al., 2022). Extensive validation studies have focused on only two mobile cognitive tests for individuals with schizophrenia: the **Jewels Trail Tests A and B** (smartphone versions of the Trail Making Test). These studies, conducted in both the U.S. and India, demonstrated the **reliability and validity** of these tests compared to lab-based assessments, despite relatively small sample sizes (18–76 participants) (Horan et al., 2024). It has been researched as an effective tool in patients with psychosis (Cohen, 2024) and college students (Patel and Torous, 2021) with anxiety or depression(Currey and Torous, 2022), among other populations.

### 3.2 Participants

Patients diagnosed with first-episode schizophrenia were recruited from the three study sites. At Beth Israel Deaconess Medical Center (BIDMC), Boston, United States, 23 individual interviews were conducted with patients who were already seeking treatment for schizophrenia at the hospital. In the focus group discussions conducted at All India Institute of Medical Sciences (AIIMS), Bhopal, India, the number of participants ranged from seven to nine per group, totaling 25 individuals. Participants were patients receiving clinical care at AIIMS Bhopal. At National Institute of Mental Health and Neuro Sciences (NIMHANS), Bangalore, India, 25 participants were recruited who were receiving clinical care at NIMHANS for a diagnosis of schizophrenia. The age range and gender distribution at each site are provided in Table 1.

**Table 1:** Summary of patient demographics.

Study Site	Number of Participants	Mean Age	Age Range	Gender		
				Male	Female	Preferred Not to Say
BIDMC, Boston	23	40	25–62	5	15	3
NIMHANS, Bangalore	25	34	25–48	19	6	0
AIIMS, Bhopal	25	34	19–50	15	10	0

### 3.3 Procedure

Eligible patients at the three sites were contacted. FGDs were scheduled at mutually convenient dates and times, and patients were compensated for their participation. The FGDs were conducted by trained researchers (SC, AR, AB, YS, EL), and standard operating protocols were put in place for any potential mental health need (e.g. feelings of distress). Written informed consent was obtained from the patients. All patients had the right to decline providing any information or leave the study at any point, without any consequences or impact on their clinical care. FGDs were moderated using the topic guide and were conducted in Hindi and English. Patients were recruited through flyers or referrals by clinicians at the sites. All sites continued with the interviews or focus group discussions until data saturation was achieved with a target sample size of approximately *n* = 25 at each site.

All interviews across the three sites followed the same format. FGDs were initiated with the discussion focused on the participant's current phone usage and experience with using health apps. Then, the FGDs transitioned to discuss the need for cognitive monitoring using smartphone apps. The interviewers then demonstrated the mindLAMP app, and all participants were shown the same slide deck on the mindLAMP app across all sites to ensure uniformity. Following the presentation, participants were allowed to explore the app through the lab study phone, answering surveys and playing cognitive games to get an immersive experience of the app. Follow up questions were asked to participants if they had any particular interest outside the scope of the questions in the guide but relevant to the development of the app.

### 3.4 Data analysis

The interviews elicited details regarding the current user experience and gathered feedback to increase user engagement. The interviews and focus group discussions were audio-recorded at all the sites, and the interviews were transcribed and coded manually. Data were analyzed following a thematic analysis approach (Braun and Clarke, 2006), which offers flexibility to identify patterns, generate codes, and organize them with broader themes. The topic guide served as the basis for the a priori themes in the framework. As the data were coded, both deductive themes were identified. At least two coders (AR, YS, EL, SC, ND) were involved in the process at all three sites. The coders were worked in the same hospital setting where the participant recruitment took place, ensuring they were familiar with the local languages, cultural nuances, and socio-cultural context of the participants. This approach helped ensure that the analysis was culturally grounded and sensitive to the participants' backgrounds. Throughout the process, themes from all sites were compared, and a finalized set of themes was agreed upon. This involved interpreting and summarizing the data, adding new themes, eliminating redundant ones, and merging overlapping themes.

## 4 Results

A key part of the thematic analysis involved identifying broader themes. These included the perceived acceptability of using the mindLAMP app in conjunction with traditional care, perceived helpfulness for improving cognition, as well as other aspects of the mindLAMP app that patients found helpful and attitudes towards engagement and usability of the app. The themes are summarized and presented with representative quotes in the following sections.

### 4.1 Theme 1 - acceptability in the context of augmenting care

Acceptability is concerned with understanding how receptive individuals are to the app and using it for cognitive assessments. The participants reflected on various dimensions, including their willingness to use the app, comfort with its functionalities, and readiness to integrate cognitive monitoring into existing routines. Concerns and reservations regarding how the app might impact traditional healthcare practices and face-to-face interactions were discussed.

The participants had a predominantly positive view of the app, expressing excitement and a willingness to use it. Many voiced their enthusiasm with comments like, *“It's pretty cool”* and *“I will feel comfortable using the app.”* When asked if they would use the app at their clinician's recommendation, nearly all participants responded affirmatively, indicating a strong acceptance. Furthermore, many were inclined to recommend the app to others, with one participant highlighting its utility for individuals struggling with memory or focus: *“I will suggest it to someone if they struggle with memory or focusing.”* Another participant stated, *“Yes, I would recommend it, independent of condition,”* underscoring the app's perceived broad applicability. Additionally, the participants also wanted content that was evidence-based and clinician-recommended, with one emphasizing, *“The exercises on the app should be such that even the doctors think that it's effective.”*

### 4.2 Limitations to acceptability

Despite the general enthusiasm, some participants expressed concerns about the app potentially replacing traditional doctor visits. These reservations were mitigated by the app being seen as a complementary tool rather than a replacement. Participants raised concerns such as, *“Yes, I would be happy to do it, but will it replace my appointments with my doctor? If it does, then I don't want to.”* Another echoed this sentiment, emphasizing the value of in-person interactions: *“Yes, I am okay with the idea, but I also like coming to the hospital and meeting you all.”* While some concerns about replacing personal interactions with healthcare providers existed, the app was largely welcomed as an adjunct to traditional care.

### 4.3 Theme 2 - perceived helpfulness of the mindLAMP app

**Fig. 1:**
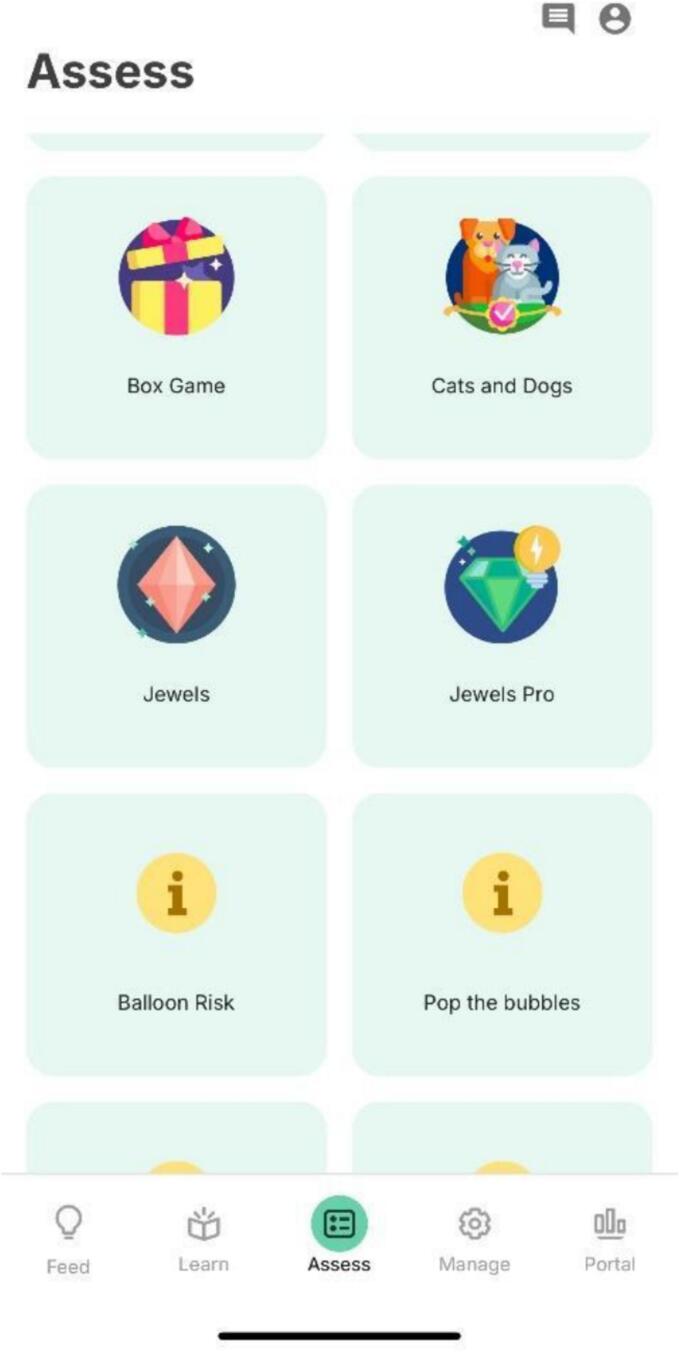
“Assess” section of the mindLAMP app.

Helpfulness of the app encompasses participants' feedback about how it helps them manage their issues, concerns, and cognitive symptoms. Participants provided rich insights into the perceived usefulness of the app, highlighting several vital aspects. Participants appreciated the cognitive tests, the content on psychoeducation and interventions, and the ability to track progress and health metrics using the Portal section of the app. A significant portion of the topic guide centered on exploring patients' perspectives on the cognitive games. Participants provided feedback, sharing insights into what they found appealing about the games in the Assess section (Fig. 1) of the app, as well as offering suggestions for improvement. This feedback, as summarized in Table 2 highlights various aspects, such as the perceived benefits of the games, areas for potential enhancement, and their overall experience with the cognitive tasks.

**Table 2:** Assessments and Feedback.

Cognitive Test	Domain Assessed	Feedback Summary
Jewels A	Attention; Working memory	• The game was well-liked and enjoyed. • The game should automatically go to the next level rather than asking if the user wishes to continue. • There could be better color contrast between the jewels and the numbers, so they are easier to read.
Jewels B	Attention; Working memory	• The game was well liked and enjoyed. • The game should automatically go to the next level rather than asking if the user wishes to continue. • The different sets of jewels should be different colors to make distinguishing them easier. • The game should display which Jewel comes next at the bottom of the screen after the first two Jewels.
Spatial Span	Working memory	• If the user selects the wrong square, the game should not advance until the correct square is selected.
Spin the Wheel	Decision making	• The game could be frustrating because victory is based on chance, leaving the user feeling powerless. • Spins went by too quickly, allotting more time would be better. • The spin count at the bottom may be somewhat small (i.e. the number was difficult to see). • The “0 won, 0 lost” roll was really dissatisfying.
Symbol Digit Substitution	Sustained attention; Processing speed	• The symbols could benefit from being colored. • Without the mapping table at all it was very difficult and frustrating.
Maze Game	Planning	• Participants reported enjoying playing this game.

Among the participants who found the cognitive tests helpful, the reasons given were related to either an improvement of cognitive function or emotive reasons. One of the participants emphasized, *“I have always liked the assess section. I have used it to track my progress. I feel like my brain is working when I play those games.”* Fig. 1 displays the Assess section in the mindLAMP app. Many participants said that they felt that it helped with their attention and focus. One said, *“I like the games; it's good focus training.”* Views such as, *“It's good. The timer made me more focused and aware. It feels purposeful.”* Another participant shared, *“I liked feeling as though the game would sharpen your mind and build your memory.”* Additionally, some mentioned benefits such as improving hand-eye coordination and mental acuity.

Patients indicated that they understand the benefits of the app in improving cognition over time, with many expressing interest in getting more explicit feedback on how the tests were helping and which cognitive domains were being targeted. Thoughts such as *“There should be more explanation for the cognitive domains the game train in more detail, what is it training, what is the benefit?”* and *“These are easy to understand and not frustrating, but I didn't understand the exact purpose of each”* emerged. One participant also commented that she would like to recommend this app to someone, *“[b]ut would want to know more explicitly what it's training. Is it focus or memory?”* Providing the users with this feedback will likely enhance the app's helpfulness.

Many participants also commented on the emotional engagement and enjoyment they experienced while using the cognitive tests. One participant described their experience, *“It served as a bit of a pastime. Like, in the evening, it provided a reason to engage with the phone, a way to pass the time and keep the mood a bit fresh.”* A participant also noted, *“If you play games in it, then a lot of time passes, and you do not feel lonely.”* One remarked, *“It keeps me busy; it's entertaining.”* A participant shared, *“I want it to be a way to relax. I actually play games that are similar [to existing apps], like Candy Crush. They are calming.”*

### 4.4 Theme 3- perceived psychosocial benefits in the MindLAMP app

**Fig. 2:**
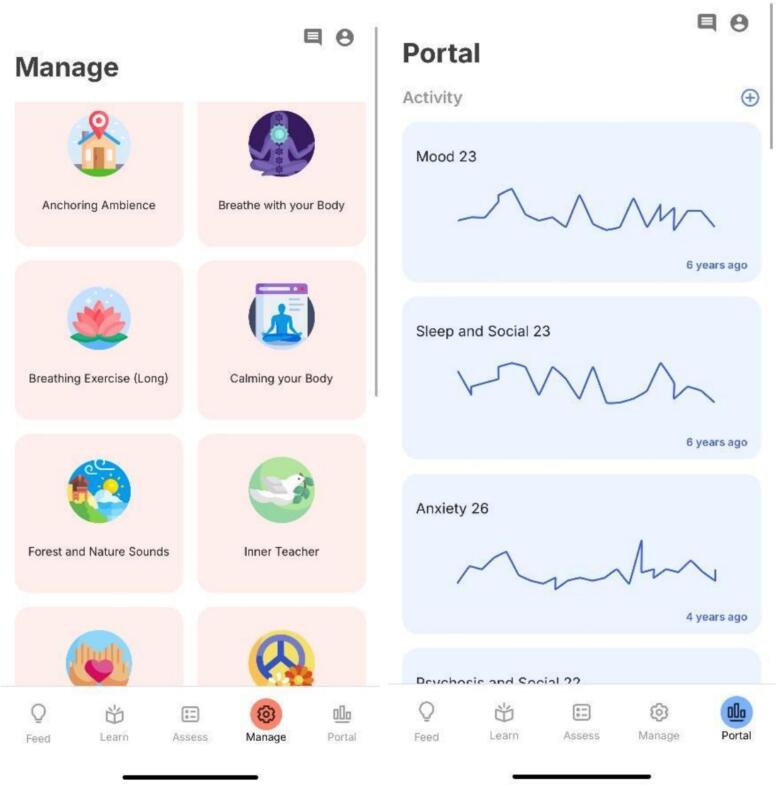
“Manage” and “Portal” sections of the mindLAMP app.

The majority of the participants found the Portal section (Fig. 2) that provides feedback to be helpful. Many saw it as a way to track their progress that could also be shared with their healthcare provider: *“If the scores stay on the app after I have answered them, I can also understand them and discuss them with my doctor when I meet him in the next follow up”*; *“I would like to see the feedback so that I can show it to my doctors.”* They also appreciated the benefits that tracking health metrics could have: *“We got to know about our wake up and sleep time, in a way we would get a reminder about at what time we sleep and wake up. Usually, we are unable to keep track of our sleep-wake routine.”* Some participants emphasized that self-monitoring mood and other health features create a sense of accountability, *“allowing for self-reflection and improvement.”*

The participants also appreciated the psychoeducation content provided on the app via the “Tips” section: *“I like this section of tips because I need stress tips, so the content was nice.”* Another participant reported, *“I feel that sometimes, you can't understand that you actually have this illness. You can't accept these things. My mom isn't able to accept what she has; she doesn't know; she doesn't feel like she has an illness. If we can see our symptoms, see that I also feel this way, we can accept that this is happening.”* Further, the mindfulness activities on the app were well received, but there was an emphasis to have audio options that are in Hindi or Kannada.

It was noted that participants sought one comprehensive app to manage various aspects of their health, particularly within the context of healthcare in India. They wanted the app to track mood, sleep, activities, water intake, diet, menses, and medication. A participant commented, *“If you can add water reminders, that would be great for me because I keep forgetting when to drink water.”* Another one said, *“If you can add the sleep feature where I can track my sleep, it will help me manage my sleep issues better.”* There were some innovative ideas for including diet plans (*“Keto diet plans also should be included in the app.”*), information on medications (*“I would like to have information about medications in it. )* and reminders (*“I'm currently getting medical treatment in the hospital, and I need a little help. I'm having trouble remembering to take my medication, so I was wondering if you could help me out with a friendly reminder.*”). Most comments for having these features in a single app came from the Indian sites, where access to care and the usage of healthcare apps were much lower compared to Boston.

### 4.5 Theme 4- improving sustained engagement

Engagement with the app is concerned with the depth and quality of user interaction. It delves into how users connect with the app, including their level of interest and the overall satisfaction derived from their experience. The participants shared feedback on barriers to sustained engagement.

Some participants shared that the cognitive tests were challenging and interesting, and they also appreciated the variety of these tests. One expressed, *“I liked that they were diverse; [the] difficulty was reasonable.”* Another shared, *“I liked that they provided a bit of a challenge.”* Some feedback about enhancing engagement with the games was also provided. Adding badges, rewards, and music or sounds to the tests was common feedback. A participant stated, *“I wouldn't mind the option for some sort of calming music in the background.”* Others suggested, *“Maybe if they had music, oriented towards focus or meditation”*; *“I would like to have sounds when spinning the wheel or pressing jewels”*; *“If they had additional sound, it could be cool, not music, but a fun ping when you press on stuff.”* A participant shared, *“It would be nice to have badges or some sort of reward, like Duolingo has characters that sing to you.”* A participant suggested, *“In the games, my suggestion is that the games should be target-related. Yeah, like Candy Crush, it is a target-related game. Similarly, the app should have target-related games.”*

Though the participants liked the app's content overall, some suggestions about making it more engaging were provided. For some participants, the content felt text-heavy, and they wanted it to be more visually engaging, to ensure focus. A participant suggested improving the app with more visual cues as they have short attention spans to go through lengthy text and images can outset boredom. Concern was also expressed about the app eventually becoming monotonous. A participant remarked, *“The design is nice, but I will get bored of the app very soon because of repetition.”* Another suggested, *“I will get bored of the games section, but if you can keep adding the games, then that will definitely help me.*”

Many participants liked how feedback was provided through graphs in the Portal section of the app (Fig. 2). A participant commented, *“I will take time to understand the scores on the app, so a graph would be nice, as I have experience reading graphs.”* Some ideas about making the feedback more comprehensible to a layman such as the patient, were provided, with one participant observing, *“This is acceptable, but adding colors and additional details could enhance its visual appeal and provide more information. By incorporating multiple colors, we can illustrate changes over time, showing our status in the initial minute compared to the following two minutes. Additionally, including percentages will offer a clear indication of our current situation.”* Although participants liked the feedback, they did not want to be overwhelmed. Suggestions to the frequency of feedback on the app were made such as having weekly averages as opposed to daily scores. While commenting on the frequency with which he would like to receive feedback, a participant said, *“Weekly assessments provide a more comprehensive overview, allowing for self-reflection and improvement. Daily updates might lead to forgetfulness regarding yesterday's status and todays, whereas a weekly format ensures better retention and understanding of progress. As for monthly results, it might be overwhelming to review them in detail. However, providing an option to view monthly results alongside weekly ones could be beneficial.”*

### 4.6 Usability

Usability is derived from how effectively and efficiently users can achieve their goals using the mindLAMP app. The participants gave feedback on elements such as the app's design, functionality, and ease of use and learning.

The participants appreciated the smooth interface and app navigation. A participant remarked, *“It is very smooth. I could go from one section to another, so it is overall very nice.”* Moreover, many of them also liked the app's display and look. Participants across all sites expressed that they liked that the app was simple, had pleasant colors, and was not very cluttered. A participant commented, *“I like the way it looks. It's not too cluttered, and it doesn't give a headache to find what's on there.”* The participants primarily thought that the app was easy to use. The consensus was that it could be used without much assistance, with a participant expressing it as *“Yes, if* [the interviewer] *didn't explain it, it would have still been easy to figure out.”*

### 4.7 Implementation of the findings added to the mindLAMP app

Based on the findings from the development phase, we have made several key modifications to the mindLAMP app for Phase 2 of the ongoing study, in which patients will use the app for a period of 30 days for pilot testing. The themes highlighted from the discussion, the importance of engagement and accessibility. Therefore, we have implemented the following changes:

1. Enhanced Visual Instructions and Scoring Information: Participants indicated that the app would benefit from more visual instructions on how to play the cognitive test and how points can be scored. In response, clear instructions for each cognitive game were added at the beginning of the test. An in-app schedule was also put in place so that participants would receive notifications for when to complete activities; this schedule was designed to reduce monotony by assigning different games each day of the week.
2. Localized Content and Audio Options: Given the multicultural context of the study, we have expanded the language options for audio instructions and mindfulness exercises in the Manage section of the app. Language support was added for Hindi and Kannada, which were specifically requested by participants in India. This localization aims to improve accessibility and user comfort, particularly in the Indian sites where language barriers were noted.
3. Ensuring Complementarity to Traditional Care: Many participants expressed concerns about the app potentially replacing traditional care. To address this, the research team at each site has clearly communicated through the informed consent form and patient interview that the mindLAMP app is a tool to supplement in-person care, and not replace it.

These modifications reflect the core feedback from our participants and are designed to optimize user engagement and usability. The upcoming pilot testing phase will evaluate the efficacy of these changes, with a focus on patient satisfaction, usability, and the impact on cognitive monitoring outcomes over the 30-day period.

## 5 Discussion

Our results are concurrent with the larger body of work done to understand smartphone apps and their relevance for people living with schizophrenia. In this work, we have shared the results from international multi-site, focus group discussions that aimed at adapting the mindLAMP app for cognitive assessments for schizophrenia. However, it is important to note that because our study includes participants experiencing first episode of schizophrenia, our findings may not be fully generalizable to the broader schizophrenia population, which includes older and potentially less technologically proficient individuals. During the FGD's users provided positive feedback on the concept and initial designs as well as suggestions for new features. While interest in the app was high, patients across sites indicated that the app should only be used to augment, not replace, in-person visits and utilized in conjunction with their clinical team. The importance of information sharing, and feedback was highlighted, focusing on improved data visualizations and actionable insights derived from the cognitive scores. These results align with the literature highlighting how apps must deliver ease of use, visual appeal, a clear interface, and engaging content as important characteristics (Alqahtani and Orji, 2020; Chawla and Saha, 2024; Jakob et al., 2022; Szinay et al., 2020; Wong et al., 2021). Similar feedback on the app was also received in previous studies conducted on the MindLAMP app (Bondre et al., 2022; Rodriguez-Villa et al., 2021).

Given that the discussion focused on the app's cognition assessments, participants offered a range of suggestions and design directions. Participants appreciated the ability of the app to change its language, icons, images, audio, and notification schedule to match their preferences. Across all sites, participants saw the cognitive assessments as more than measurements and felt the nature of these assessments offered a high degree of gamification. Across the different cognitive domains, participants felt the Jewels A and Jewels B were the most engaging games. This was seen as an advantage as it would increase engagement, which is important given that low engagement is often an Achilles heel of digital health efforts today (Philippe et al., 2022).

Participants noted these cognitive tests could also be useful for attentional redirection when needed and therefore serve a therapeutic benefit. While the cognitive tests in the mindLAMP app are not designed to be therapeutics, other apps that have offered distraction have demonstrated that they can decrease the frequency and intensity of auditory hallucinations common in schizophrenia (Jongeneel et al., 2024). While the actual therapeutic potential of the app is unknown, assessing clinical measures of psychosis when testing the app will be useful to better understand this potential secondary benefit of use.

Providing users with detailed feedback on the cognitive domains targeted by each test, along with the associated benefits, could significantly enhance the perceived utility of the app and contribute to more meaningful engagement with the cognitive assessments. These options allowed for the participants to have an option to engage in psychosocial interventions on their phone and have greater engagement in the app. Across all sites, participants reflected on the importance of the app being used in conjunction with their care and a clinical team that endorses the app. Standard operating protocols of integrating such apps in clinical care should have the clinician demonstrate the cognitive tests in person and allowing participants to practice under supervision would enhance their understanding of proper usage and the relevance of the tests. Participants were cognizant that there are already a plethora of digital health apps and that most of them, especially those publicly accessible and for schizophrenia, have not proven helpful (Kwon et al., 2022). As a result, they emphasized the need for clinician-endorsed, evidence-based apps to ensure their utility. Additionally, there was a concern that the mindLAMP app might be used to minimize their time with a clinician with participants expressing a preference for their use in a collaborative, integrated hybrid care model. This theme aligns with the growing evidence base towards hybrid models, where technology is employed as a continuity of care (Balcombe and De Leo, 2021). Although the discussions did not specifically focus on passive (sensor) data collection that the app can collect to provide context for cognition (eg sleep or step count), users did not raise privacy or ethical concerns. Participants conveyed reliance on the clinician to make informed decisions to suggest the use of the app in context of care, trusting that the information would be utilized to improve their symptoms.

While the main themes were consistent across sites in India and the US, there were certain differences between the sites. Access to and use of healthcare apps is more ubiquitous at the Boston site than at the sites in India, especially Bhopal. Thus, users at Bhopal, which is a smaller urban setting relative to Bengaluru and located in predominantly rural state in central India, wanted the app to offer more services beyond cognitive assessments. While outside the scope of the current project, this is feasible as the mindLAMP platform can support expanded services delivery and clinical operations (Macrynikola et al., 2023).

Site-specific differences also emerged regarding concerns about the digital divide. While the recruitment strategy for the study creates a bias towards individuals with some digital literacy, participants from Bhopal expressed more concerns about the digital divide than those from the larger urban settings of Boston and Bangalore. The digital divide concerns the disparity between individuals who can effectively utilize new information and communication technologies, for instance, the Internet, and those who cannot (Gunkel, 2003). This divide has also been conceptualized as a continuum (Katz, 2017), with factors like age, gender, education, geography, and income impacting where an individual stands (Elena-Bucea et al., 2021). Participants at the Bhopal site specifically highlighted concerns about internet access and helping others within their community acquire smartphones. Although the mindLAMP app functions offline or in airplane mode, it still requires internet connectivity to download and transmit data, which creates a barrier to those who do not have consistent internet access. As a result, ensuring there is a digital equity plan for the deployment of the software was raised as an important issue for larger implementation efforts.

## 6 Conclusions

This international focus group guided the design and deployment of smartphone-based cognitive assessments in schizophrenia on the mindLAMP platform. Participants shared positive opinions and suggestions for more visualizations but cautioned that the app should be used in the context of ongoing care and not to replace in-person visits. Creating an ecosystem of services around digital health technologies is a productive approach our team can support as we continue to expand this research and related efforts.

## CRediT authorship contribution statement

**Aishwarya Raje:** Writing – review & editing, Writing – original draft, Data curation, Conceptualization. **Abhijit R. Rozatkar:** Writing – review & editing. **Urvakhsh Meherwan Mehta:** Writing – review & editing, Writing – original draft. **Ritu Shrivastava:** Writing – review & editing. **Ameya Bondre:** Writing – review & editing, Writing – original draft. **Manaal Amir Ahmad:** Writing – review & editing. **Anshika Malviya:** Writing – review & editing. **Yogendra Sen:** Writing – review & editing. **Deepak Tugnawat:** Writing – review & editing. **Anant Bhan:** Writing – review & editing. **Tamonud Modak:** Writing – review & editing. **Nabagata Das:** Writing – review & editing. **Srilakshmi Nagendra:** Writing – review & editing. **Erlend Lane:** Writing – review & editing. **Juan Castillo:** Writing – review & editing. **John A. Naslund:** Writing – review & editing, Writing – original draft. **John Torous:** Writing – review & editing, Writing – original draft, Conceptualization. **Soumya Choudhary:** Writing – original draft, Formal analysis, Conceptualization.

## Ethics approval

**Letter Of Permission: AIIMS Bhopal:** EL0072;**NIMHANS IRB no-** NIMHANS/41st IEC (BEH.SC.DIV)/2023; **Sangath**- AB_2023_88.

## Funding

This research was funded by grants from 10.13039/100010269Wellcome Trust (UK) with the grant number GRT65216.

## Declaration of competing interest

The authors declare no conflicts of interest.

## Appendix 1 FGD guide for patients

**Section A General- Phone/digital app use**

a. Phone use

1. What type of smartphone do you have access to?

2. If you have access to a smartphone, how do you access it? (own, shared, etc.)

3. What are the challenges you face while using a smartphone? (network issues, power issues, etc.)

b. App use
  1. Which apps do you often use on your phone? When do you use them? How much time do you spend using them? Why do you like them? Any apps you don't like / often use?
  2. Do you remember why you started using them (pick a favorite one) to begin with?
  3. How did you learn to use these apps? How long did it take for you to become familiar?
  4. How do you decide whether to continue using an app or uninstall it?
c. If you don't use apps, why do you not? What prevents you from using apps?

**Section B Apps for health Care**

1. Have you used any health-related apps?

2. Has a doctor ever recommended an app to you? If yes, why?

3. Did you use/like them?

4. Has any app contributed to improving your mood/functioning/cognition? If so, which app and what did you notice?

5. Which areas do you think an app would help? (Sleep/Medication adherence/Stress/Anxiety/Connecting with peers and support/Distractions (games)/Mood tracking (journaling), or Cognition).

6. Do you use any apps to access healthcare? If not, why not?

i. If yes, which app à How and why did you choose it? How often do you use it? What in particular does it help you with, or its most useful features?

**Section C Feedback on mindLamp Cognitive tests**

1. First impression/What did you think? What do you like or dislike? Do you like the way it looks? Is it easy to use?

2. How do you like the overall idea of using an app for testing your mental health condition (cognitive tests) in a non-hospital environment?

3. How do you like the idea of using mental health assessments (cognitive tests) through digital platform or app?

4. How would you want this cognitive testing data to be shared back with you (eg scores, charts, discussion).

5. What is your feedback on the design of the mental health assessments (cognitive tests) delivered over smartphone apps?

6. What is your feedback on the content of mental health assessments (cognitive tests) delivered over smartphone apps?

7. Do you think that mental health assessments (cognitive areas) provided in the app are sufficient and relevant?

8. What else or more mental health assessments (cognitive areas) could or should be covered in these tests?

9. What do you think of the practicality of these mental health assessments (cognitive tests) in the app? (e.g., number and types of tests in a day or a week).

10. What features or functions of apps related to mental health assessments (cognitive tests) feel more relevant to you? Why?

11. Would you feel comfortable using the app for mental health assessments (cognitive testing)? Why or why not?

12. What are the features and functions in general do you like in the mindLamp app?

13. Any other suggestion/recommendation?
